# Under-reporting of greenhouse gas emissions in U.S. cities

**DOI:** 10.1038/s41467-020-20871-0

**Published:** 2021-02-02

**Authors:** Kevin Robert Gurney, Jianming Liang, Geoffrey Roest, Yang Song, Kimberly Mueller, Thomas Lauvaux

**Affiliations:** 1 School of Informatics, Computing, and Cyber Systems, Northern Arizona University, Flagstaff, AZ USA; 2 ESRI, Redlands, CA USA; 3 National Institute for Standards and Technology, Gaithersburg, MD USA; 4 Laboratoire des Sciences du Climat et de l’Environnement, Gif-sur-Yvette, France

**Keywords:** Climate-change mitigation, Climate-change impacts, Energy management

## Abstract

Cities dominate greenhouse gas emissions. Many have generated self-reported emission inventories, but their value to emissions mitigation depends on their accuracy, which remains untested. Here, we compare self-reported inventories from 48 US cities to independent estimates from the Vulcan carbon dioxide emissions data product, which is consistent with atmospheric measurements. We found that cities under-report their own greenhouse gas emissions, on average, by 18.3% (range: −145.5% to +63.5%) – a difference which if extrapolated to all U.S. cities, exceeds California’s total emissions by 23.5%. Differences arise because city inventories omit particular fuels and source types and estimate transportation emissions differently. These results raise concerns about self-reported inventories in planning or assessing emissions, and warrant consideration of the new urban greenhouse gas information system recently developed by the scientific community.

## Abstract (web summary)

Many cities in the US self-report greenhouse gas emissions. Here, the authors find that US cities under-report their own greenhouse gas emissions, on average, by 18.3% because city inventories omit some fuels and source types and estimate transportation emissions differently.

## Introduction

Almost three-quarters of fossil fuel carbon dioxide (FFCO_2_) emissions, the most important anthropogenic greenhouse gas (GHG), emanate from cities^1^. Projections show cities could add over two billion people this century with global urban area tripling by 2030^2,3^. This outsized role is compounded by the concern that urban development can lock-in resistance to low-carbon pathways through technological, institutional, and behavioral inertia^4^. At the same time, cities are taking a leadership role in climate emissions mitigation activities with cities across the globe pledging to ambitious emissions mitigation goals and playing an increasingly important role in the international climate change negotiating process^5^. A necessary element in urban emissions mitigation is the development of a numerical accounting of GHG emissions following a standardized method^6^. This enables emissions mitigation planning activities and the establishment of emissions baselines with which to establish reduction targets. Cities across the globe have developed estimates of their GHG emissions, which we refer to as “self-reported inventories” (SRIs), mostly following one of a few publicly available protocols^7–9^. However, there is no systematic, peer-reviewed assessment of SRI quality or accuracy in spite of their importance to establishing baseline emissions, urban mitigation target setting, and mitigation outcome assessment.

Given this assessment gap, we have investigated 48 SRIs published online by cities in the USA and compare these to urban extractions from our recently completed, research-driven Vulcan version 3.0 estimate of US fossil fuel carbon dioxide (FFCO_2_) emissions. Vulcan quantifies complete FFCO_2_ emissions at points (e.g., factories, powerplants), lines (i.e., roadways), and polygons (i.e., US census block-groups) for the whole US spanning the 2010–2015 time frame using a large number of publicly available input datasets^10^. The data inputs are primarily archived from local fuel, activity, and flux monitoring data. The Vulcan v3.0 results have been compared to an emissions estimate based on observations of ^14^CO_2_ in the USA, an ideal atmospheric tracer for FFCO_2_, and is within 1.5% of the atmospheric-based result^11^. Furthermore, the urban-intensive INFLUX effort in the city of Indianapolis shows Vulcan results within 3% of atmospheric-based results over a 3-year mean timespan, and within 6% annually^12^.

The samples of 48 cities included in this comparison were chosen through a combination of previously published work^13^, the presence among the top 100 emitting cities in the USA, and the availability of adequately documented SRI results. The 48 sample cities account for 13.7% of 2015 US urban emissions and 17.7% of 2015 US urban population. Unambiguous city government boundaries were used to extract sector/fuel-specific FFCO_2_ emissions from the “native” (points, lines, polygons) Vulcan FFCO_2_ emissions. Both the Vulcan and SRI results were inspected to ensure geographic alignment of city boundaries and emission sectors, gases, and scope. For example, were individual emission sectors (e.g. airport, industrial) absent in the SRIs, they were similarly eliminated in Vulcan, to the extent SRI documentation offers clarity on sector representation. The omission of sub-sector elements in the SRI (e.g., individual fuels within a sector, individual factories in the city domain), rarely documented, are considered part of the comparison in this analysis, and hence not adjusted for (see “Methods”).

## Results

**Fig. 1:**
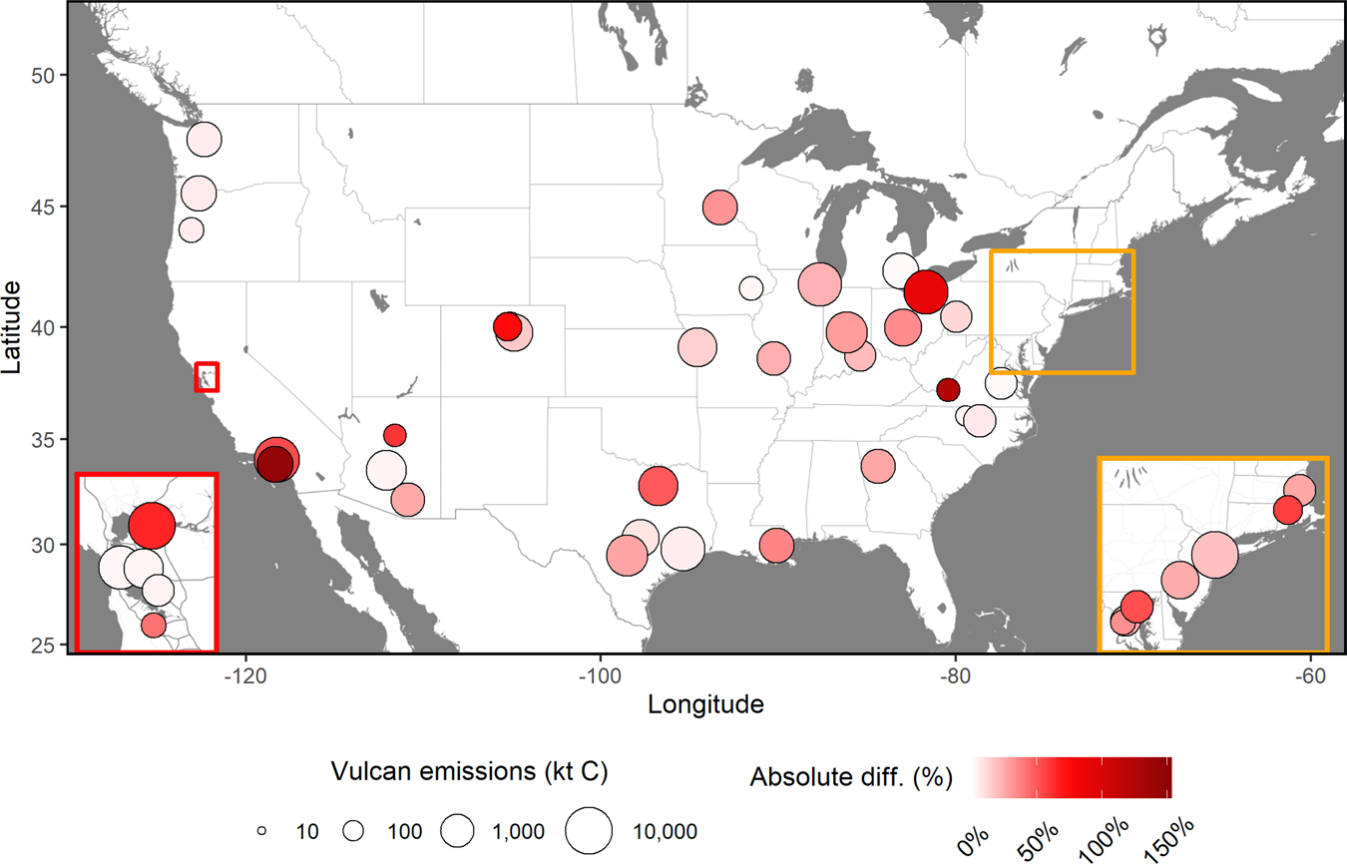
Total individual city (*N* = 48) FFCO_2_ emissions and absolute difference (AD = positive RD) between the Vulcan version 3.0 data product and self-reported inventories (SRIs). AD values denoted by color scale and total emissions denoted by circle size. Two regions are expanded (California Bay Area and the Northeast coastal urban corridor) for closer inspection (see “Methods” and Source data for additional details). Background map tiles by Stamen Design (no changes made), under CC BY 3.0, data by © OpenStreetMap contributors, under ODbL.

The mean relative difference (RD) between the two emission datasets is +18.3% (Vulcan > SRI; calculated as [(Vulcan – SRI)/average(Vulcan, SRI)]) with a mean absolute (unsigned) relative difference (MAD) of 29.1% (Fig. 1). This is in the context of a mean RD 95% confidence interval of +5.2%/+31.7% for the Vulcan urban emissions. Of the cities reporting fewer emissions than Vulcan (*N* = 37), the mean RD value is +30.7%. Furthermore, the summed difference across all 48 cities in units of emitted mass per year is 19,076,760 tC/year, a value nearly equivalent to the 2015 Massachusetts state emissions (Table 1). Were the 18.3% difference extrapolated to all US cities, the total would be 129,219,255 tC/year (Source data), an amount 23.5% larger than the entire 2015 California state emissions. We find a smaller RD value in the Western half of the USA (+11.0%) compared to the East (+25.0%), but a nearly identical MAD value (30.3% and 28.0%, respectively) due to the greater number of negative differences in the Western cities, causing some cancelation of the positive differences. Finally, we find little correlation (Pearson’s *R* = 0.01) between the individual absolute relative difference (AD) values and the magnitude of city emissions.

**Table 1:** State total FFCO_2_+ cement production emissions for the year 2015 as generated by Vulcan version 3.0^10^.

State	Total emissions (tC)
AK	7698510
AL	42704041
AR	16561519
AZ	29782799
CA	104660731
CO	31918346
CT	10610878
DC	1230078
DE	4445990
FL	78340164
GA	42264025
HI	4956370
IA	21843909
ID	6694903
IL	67373603
IN	55895053
KS	19782874
KY	35159975
LA	47294753
MA	20694772
MD	19458650
ME	5359411
MI	49813475
MN	33056713
MO	37477007
MS	18315636
MT	8950523
NC	42308189
ND	13208952
NE	14012960
NH	4360434
NJ	26118536
NM	14628211
NV	12791132
NY	53255701
OH	68698200
OK	30469338
OR	11373707
PA	70973620
RI	5099102
SC	22082573
SD	3604415
TN	28900311
TX	151055606
UT	20803673
VA	28278324
VT	1839216
WA	20867652
WI	31991556
WV	23249400
WY	21966262
US	1544281777

**Fig. 2:**
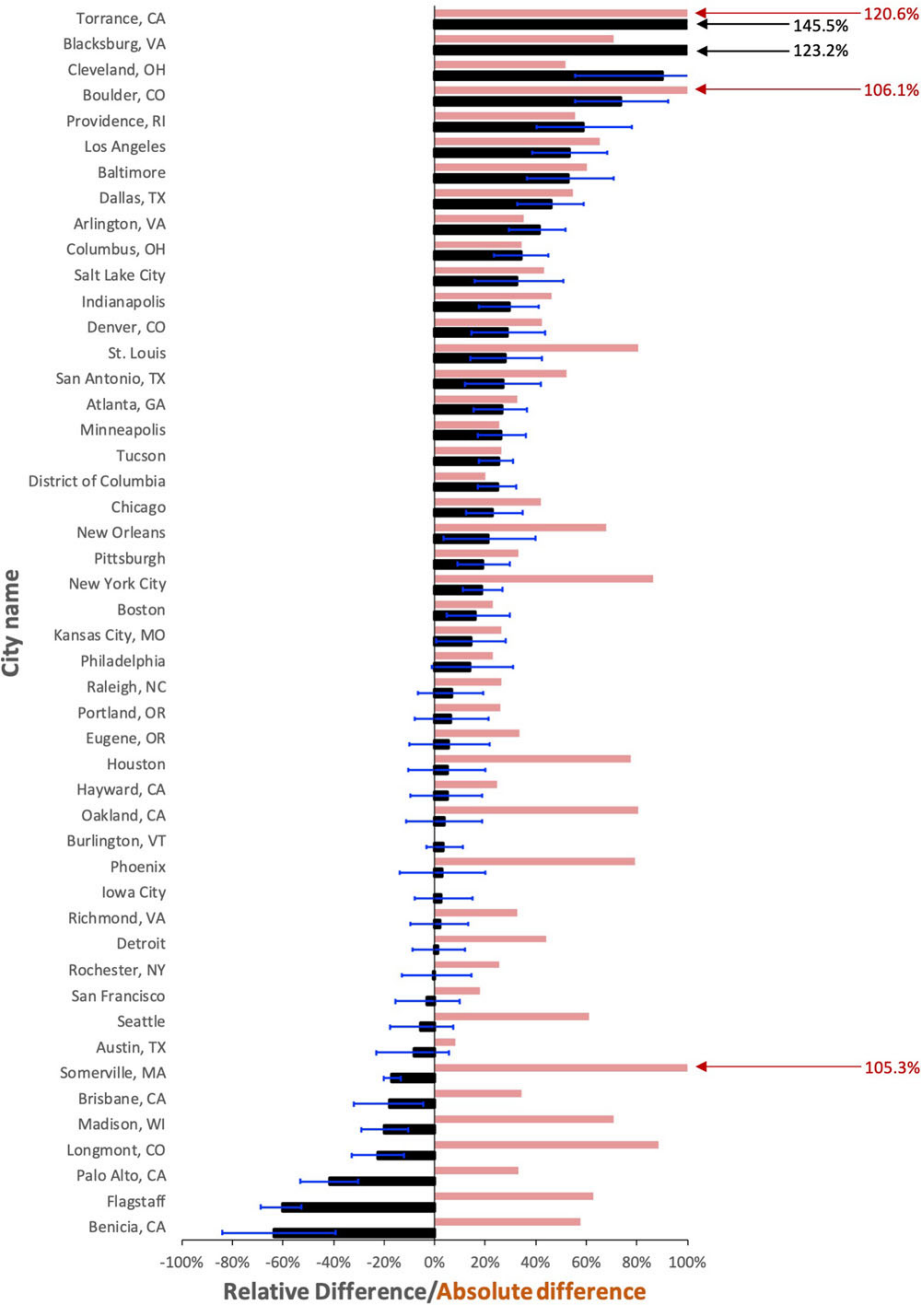
Sector-based individual city (*N* = 48) FFCO_2_ emissions relative difference (RD) between the Vulcan version 3.0 urban FFCO_2_ emissions and self-reported inventories (SRIs). Black: total emissions RD; error bars: total emissions RD range from Vulcan 95% confidence interval; red: mean across-sector absolute relative difference (MASAD). RD calculated as [(Vulcan – SRI)/mean(Vulcan, SRI)]. Scale capped at 100%. Not all sector categories available in all cities; where individual sectors were missing in the SRIs, the mean only includes those available in both Vulcan and SRI results (see “Methods” and Source data for additional details).

**Fig. 3:**
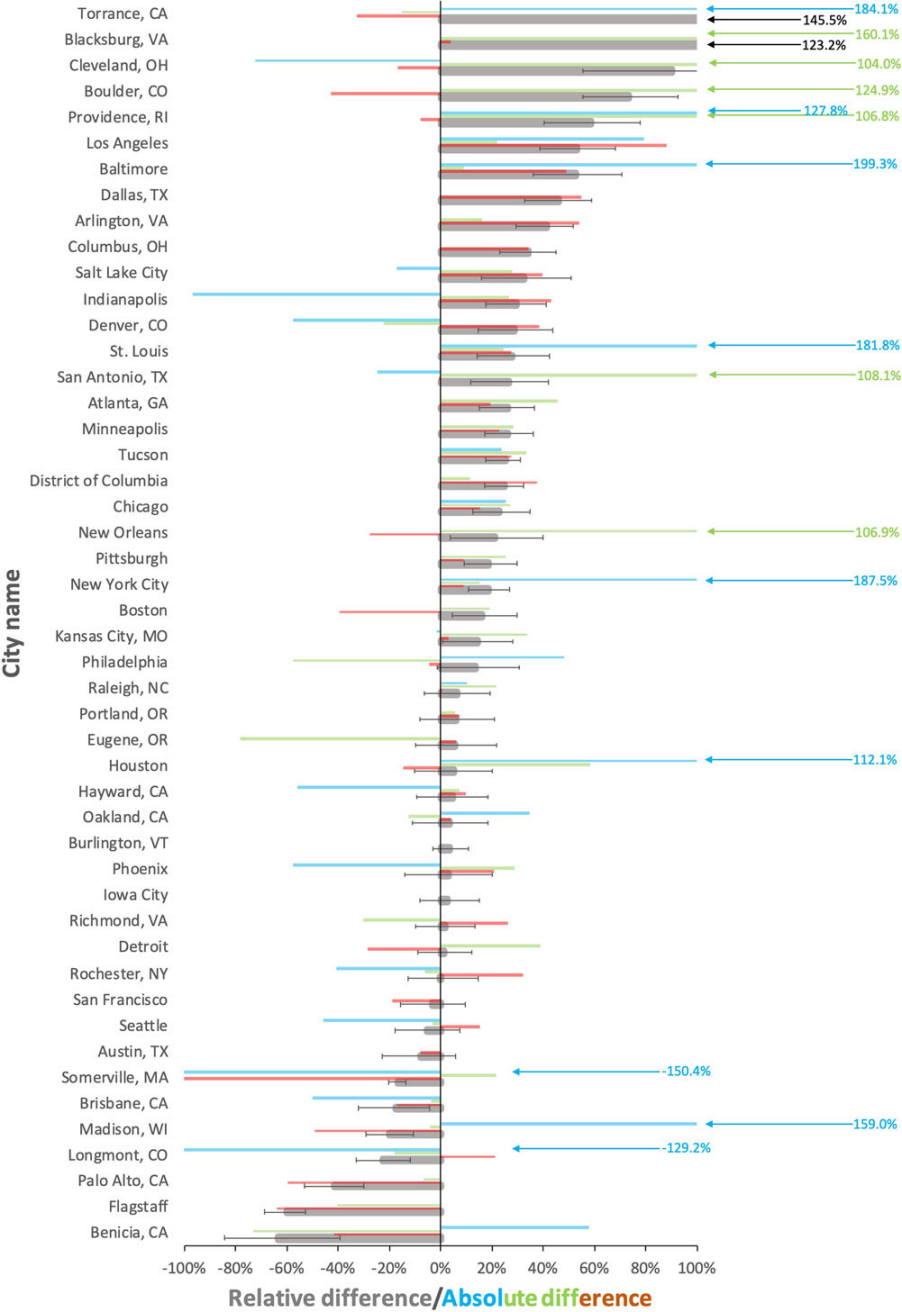
Sector-based individual city (*N* = 48) FFCO_2_ emissions relative difference (RD) between the Vulcan version 3.0 urban FFCO_2_ emissions and self-reported inventories (SRIs). Black: total emissions RD; error bars: total emissions RD range from Vulcan confidence interval; red: onroad emissions RD; green: stationary (residential + commercial + industrial) emissions RD; blue: other transportation (airborne + marine shipping + nonroad + railroad) emissions RD. RD calculated as [(Vulcan – SRI)/average (Vulcan, SRI)]. Scale capped at 100% RD. Not all sector categories were available in all cities; where individual sectors were missing in the SRIs, the aggregate category only sums those available in both Vulcan and SRI results (see Source data for additional details).

Figure 2 provides the individual city RD values for the total emissions and the mean across-sector AD (MASAD) in each city. The mean of these MASAD values for all cities is 50.3%. This demonstrates that even for cities with close agreement in total emissions, individual sectors show large discrepancies. For example, the city of Detroit has a total RD of +1.3%, but a MASAD value of 44.2% comprised of a RD of −28.5% for onroad sources and a compensating RD of +38.7% for stationary sources (Fig. 3). The mean of the sector-specific AD values across all cities for the onroad (*N* = 46), stationary (*N* = 43), and other transportation (*N* = 27) sectors is 28.1%, 37.9%, and 82.6%, respectively (Fig. 3). However, for those cities reporting fewer emissions than Vulcan, the differences are 25.7% (*N* = 28), 44.9% (*N* = 23), and 102.2% (*N* = 11), respectively.

Careful examination of the differences between Vulcan v3.0 and the SRIs suggests that the most common differences are related to SRI omission of petroleum fuel use and point source emissions in the industrial/commercial sector, different accounting perspectives on marine shipping and airborne emissions, and different methods in onroad emission estimation. Many cities collect utility natural gas consumption data on sector-based stationary sources. These data will oftentimes miss petroleum consumption occurring in buildings typically associated with industrial point source processes or smaller fuel supply contracts that are not provided by a centralized utility. SRIs will often isolate the component of marine shipping to local activity in harbor areas or attempt to estimate the resident population utilization of airborne activity. Cities also estimated onroad emissions variously using gas station fuel sales or travel demand modeling to isolate onroad emissions stimulated by activity within the city. Vulcan, by contrast, includes all emission sources within the administrative city boundary, including airborne emissions from taxi/takeoff/landing up to 3000 ft above ground, all marine shipping emissions in waters within the city boundary, and emissions from all roadways within the city regardless of the origin/destination of travel. Further explanation of the individual city/sector differences are difficult to isolate with the SRI information provided. An important caveat to this study is that better documentation of SRI system boundary details could change the numerical difference statistics found here.

## Discussion

The development of SRIs was a rational response to the need by cities to tackle GHG mitigation, a necessary and globally relevant effort, given the large role cities play in emissions and their governance purview over many aspects of the urban emission landscape. However, constructing baseline emissions and objective tracking over time using city SRIs raises several challenges, and these are likely responsible for the differences found in this study. The development of an SRI is a costly endeavor, placing a burden on city staff and resources. Data collection, processing, and modeling can present technical challenges for cities, further burdening available resources and often resulting in incomplete estimates or biased outcomes, as demonstrated here. Compounding the problem, independent objective assessment of SRI estimates creates additional technical hurdles and is thus, rarely attempted. These challenges are particularly important when placed in the context of the reduction targets. For example, the city of Indianapolis has indicated that they aim to make a 20% reduction in building GHG emissions between by 2025 relative to 2016 values^14^. However, with the 26.9% underestimate found here, it will be difficult to know when and if this target is truly achieved or track progress towards it.

A potential solution to this challenge is a more systematic GHG flux information system that combines the type of bottom-up quantification exemplified by the Vulcan data product used here with a tiered (ground, airborne, space-borne) atmospheric observation and model system^15^. Rather than being independent of individual city efforts, building and managing a GHG flux information system will require collaboration with city staff, availing of local knowledge and tailored to local needs. An urban-scale prototype of such a system has been piloted in the city of Indianapolis through the INFLUX experiment with similar prototypes being developed at the national scale^11,12^. This approach could offer a comprehensive, consistent approach across the entire continental landscape delivering fine spatial (e.g., down to the neighborhood/street scale) and functional (e.g., sector, sub-sector, combustion process) detail for better targeting of mitigation policy^16^. It also offers the ability to track mitigation progress using the most accurate arbiter of impact, atmospheric GHG amounts. Finally, an advantage of these approaches is their ability to conserve mass through spatial scales, allowing for consistency, comparability, and context from the nation to the building.

Accuracy and precision are critical to estimating GHG emissions, whether reported by a city, state, or country. The absence of an accurate emissions assessment (i.e., baseline and ongoing) makes prioritizing mitigation policy options difficult, can lead to misallocation of scarce mitigation resources, and presents challenges to independent assessment and course correction. The results presented here raise serious concerns about the current self-reported approach to quantifying urban GHG emissions in the USA. Similar dynamics may be at play in cities across the globe where SRIs have similarly been reported^17^. Fortunately, there is progress on building a systematic emissions quantification system that promises a systematic approach to generating space/time-resolved, atmospherically calibrated emissions information for all cities in collaboration with local authorities. With such a collaborative system, urban GHG mitigation practitioners can devote time and resource to the activity they have the greatest knowledge and political influence over: the best mitigation strategies for their city.

## Methods

### Cities analyzed

The cities compared in this study were collected according to a combination of available literature, emissions magnitude, and adequecy of documentation. A recent paper by Nangini et al. provides a list of cities that have completed SRIs, reporting them to the Carbon Disclosure Project (CDP)^18^. This list of 64 US cities was combined with the top 50 urban emitters from the 2011 Vulcan estimate. A systematic online search was performed to locate documents that reported SRI results for each of these cities. Many cities did not have published inventories or the inventories were published but the information content was insufficient to isolate those emissions comparable to the Vulcan results. Furthermore, SRI estimates were not considered if they reported for years outside the Vulcan timespan (2010–2015). Four exceptions were made for cities with emissions reported in years very close to the Vulcan timespan: Columbus, OH: 2017; New Orleans, LA: 2007; Longmont, CO: 2016; Salt Lake City, UT: 2009. In these cases, the year closest to the Vulcan timespan (2010 or 2014) was used. The sensitivity of the results to the removal of these four cities was negligible with little impact on the mean statistics (Table 2).

**Table 2:** Standard case and sensitivity of statistical results to removal of four cities with SRIs outside the 2010–2015 timespan of the Vulcan output (Columbus, OH: 2017; New Orleans: 2007; Longmont, CO: 2016; Salt Lake City, UT: 2009) and to the division of the Eastern US versus the Western US.

Sensitivity cases	No. of cities	Mean RD (%) [stdev]	MAD (%)	Mean RD of positive diff (%) [*N*]	Mean RD with 95% CI
Standard	48	+18.3 [38.1]	+29.1	+30.7 [37]	+5.2/+31.7
Remove four cities	44	+18.5 [39.2]	+29.2	+30.8 [34]	+5.4/+31.8
Eastern US	25	+25.0 [31.2]	+28.0	+30.1 [22]	+12.8/+37.8
Western US	23	+11.0 [44.0]	+30.3	+31.6 [15]	−3.0/+25.1

The division of East/West was placed at a longitude of −94.0°. See Source data for additional detail.

**Fig. 4:**
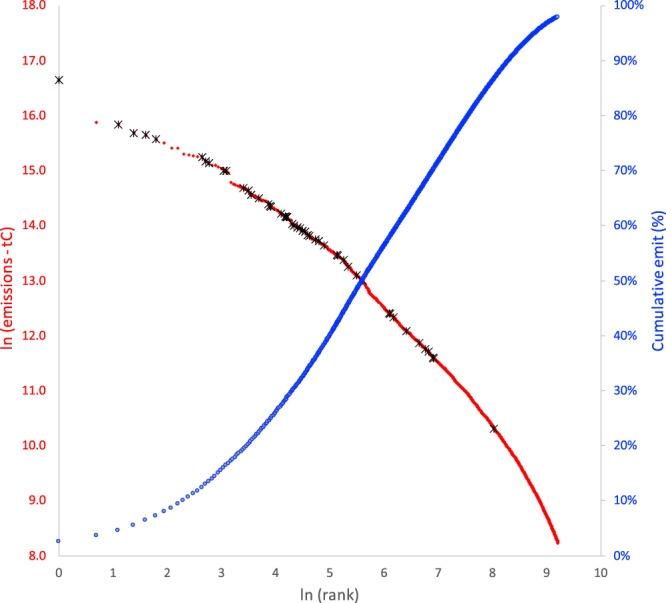
Rank distribution (red) and cumulative emissions (blue) of US city FFCO_2_ emissions. The sample of 48 cities included in this study are denoted with black “X” within the rank distribution. City FFCO_2_ emissions are from the Vulcan v3.0 results within US Census Designated Places. The sample of 48 cities used for comparison represents 13.7% of 2015 US urban FFCO_2_ emissions and 17.7% of the 2015 US urban population. Of these 48, 28 cities are within the top 100 urban emitters in the USA (see Source data for additional details).

The emissions ranking of the final list of 48 cities in relation to a rank distribution of all cities in the USA, in addition to their share of cumulative urban emissions, is shown in Fig. 4.

### Urban boundaries

To align the urban boundaries in the annual Vulcan version 3.0 results to the city boundaries in the SRIs, we use the US Census Bureau Designated Places (DPs) reflecting boundary conditions in the year 2010^19^. Online SRI documentation rarely defines the precise boundary of the domain considered but it is assumed that the SRI emissions reflect those of the stated city within the governance boundary, unless otherwise described. The US Census DP for the 48 cities considered in this study map exactly to a Google map boundary search for the named cities. The only exception relates to coastal cities in which the DP boundary will extend offshore, while cities may only include emissions from the land area within their administrative boundary. This will create differences in the marine shipping sector.

The Vulcan emissions were regridded at 300 m × 300 m so they can be more finely attributed to the DP entities. The parameters of the 300 m grid are shown in Table 3. The DP shapefile, containing 28,705 polygon features, each representing a DP entity, was rasterized using ArcGIS into the same 300 m × 300 m Vulcan grid. In rasterizing the DP polygons, each grid cell was assigned a DP identity from the polygon that overlaps the center of the cell (https://pro.arcgis.com/en/pro-app/tool-reference/conversion/polygon-to-raster.htm). The 300 m × 300 m gridded Vulcan output was then aggregated by DP entity to generate the emissions values for the 28,705 DP entities. The caveat of this method is that certain small, localized sources (e.g., point sources) may fall within a grid cell associated with a certain DP entity, even if the actual point source falls just outside of the urban boundary polygon. Likewise, it is possible that localized sources located just within the urban boundary polygon may be omitted if the DP entity is not assigned to a grid cell that only partly overlaps the polygon. This is minimized by the fact that many city boundaries are located in less dense portions of an urban area.

**Table 3:** Parameter values for the 300 m grid used to intersect Vulcan emissions with urban boundaries.

Parameter	Value
Datum/ellipsoid	WGS84
Projection	Lambert Conformal Conic
Dimensions	9551 rows × 15,340 columns
Resolution	300 m
Extent	xmin = −2,272,425, xmax = 2,329,575, ymin = −1,589,775, ymax = 1,275,525
Longitude of origin	97.0°W
Latitude of origin	40.0°N
Reference latitude 1	33.0°N
Reference latitude 2	45.0°N

Both the US Census Bureau DPs and the shapefiles underlying the Vulcan v3.0 emissions were gridded into a 300 m grid. Vulcan emissions were then aggregated by DP entity to generate emissions for each designated place.

A population value was also assigned to each DP entity following the same method with the US Census Bureau 2010 population data^19^.

### Dataset alignment

Examination of the 48 SRI results made clear that a direct comparison of the Vulcan output to the SRI output would suffer from a series of categorical mismatches. Hence, we proceeded with a number of adjustments to align the results for comparison. The first of these was to ensure consistency in scope. Three scopes have been identified in the literature: scopes 1, 2, and 3^20,21^. When applied to FFCO_2_ emissions, scope 1 reflects emissions that physically emanate from fuel combustion within the geography considered (i.e., city administrative boundary). Scope 2 refers to FFCO_2_ emissions resulting from the consumption of electricity occurring within the boundary of the city, regardless of where the emissions physically occur. For example, a given city may have one power production facility within city limits. City residents may consume electricity at such a level as to use all electricity at that in-city facility and electricity from an additional power production facility outside city limits. Scope 1 emissions in the electricity production sector, in this example, would be comprised of that single facility. Scope 2 emissions, by contrast, would include the emissions from both facilities in proportion to the electricity consumed within the city limits. Finally, scope 3 emissions allocate the emissions resulting from the production of goods and services, regardless of where they occur, to the point of consumption. For example, were a factory in our example city producing goods that are exported to consumers outside city limits, those emissions would not be accounted for within the example city, but allocated to the locations where final consumption occurred. Conversely, the quantity of emissions necessary to produce and transport goods to the example city consumers would be counted as within-city emissions.

Vulcan quantifies all CO_2_ emissions from the combustion of fossil fuels and cement production within the geographic boundary of the city domain or scope 1 emissions. Vulcan does not include biogenically originated emissions, fugitive emissions, or non-CO_2_ GHGs. Because none of the SRIs examined for this study included emissions from cement production (stoichiometric emissions as opposed to fossil fuel combustion used to process cement production), these emissions were removed from the Vulcan results.

The reason that the Vulcan FFCO_2_ emissions data product was initially restricted to scope 1 emissions was the use of Vulcan within systems that utilize atmospheric mixing ratios measurements to infer surface fluxes, often referred to as the atmospheric “inversion” approach^22–24^. Vulcan and other space/time-explicit emissions data products are often used as a boundary condition or “prior” flux to these systems. The inversion systems use models of atmospheric motion to invert atmospheric mixing ratios over a given spatial domain, and hence the surface flux boundary condition or prior flux must reflect fluxes specific to the geography considered—scope 1 emissions only.

Scope 2 and 3 are of understandable interest to urban GHG practitioners. However, to avail of the independent estimate of fluxes that atmospheric inversion systems can provide, scope 1 emissions (which typically comprises the largest emissions among the scope perspectives) are important to isolate within the overall emissions accounting. Independent assessment using atmospheric inversions offers an important element in evaluating inventory estimates and course-correcting mitigation activity.

The city SRIs, in contrast to the Vulcan approach, often will include elements of scopes 1, 2, and 3 combined to comprise the total city inventory. The mixture of scopes is often a reflection of individual city interests and partly a reflection of the protocols currently used by cities to estimate emissions. For example, Vulcan quantifies all FFCO_2_ emissions from powerplants residing within the city boundary regardless of where the electricity is sold (scope 1). Most cities, however, will estimate the FFCO_2_ emissions driven by consumption of electricity within the boundary of the city, regardless of where those emissions physically occur (scope 2).

In light of these differences, each city SRI was carefully examined in order to isolate the scope 1 sector emissions. Where scope 1 emission sectors were absent in the SRIs, they were similarly eliminated in Vulcan. Typical sectors missing in the SRIs were airport, railroad, and nonroad. Vulcan currently does not report emissions associated with the decay of material in the waste stream, and hence this was removed from the SRIs. Removal of the emissions associated with waste also assists in more closely aligning the emitted gases. Most of the city SRIs report in units of equivalent CO_2_, which incorporates non-CO_2_ GHGs weighted by their relative influence on climate change. Since the non-CO_2_ emissions in the SRIs are dominated by waste processing and Vulcan only quantifies CO_2_ emissions from fossil fuel combustion. Nevertheless, there may be a small amount of non-CO_2_ emissions within the SRIs that are not quantified in Vulcan and we would, therefore, expect the SRI results to report larger emissions than Vulcan, all else being equal.

The average proportion of the individual city emissions after these alignment steps relative to the original total was 86.4%, indicating that the comparison carried out here captures most of the original SRI emissions. All of the individual adjustments to both the Vulcan and SRI results are described in Source data.

### Comment on Nangini et al.’s study

Nangini et al. reported on an archive of SRIs that, in the USA, were submitted to the CDP^13^. These SRIs are supplied to the CDP directly with little documentation or information support. Nangini et al. performed some quality control on the submitted data and appended the SRI submissions with a variety of socioeconomic ancillary data (e.g., population, gross domestic product, climate). The paper was primarily aimed at documentation support for the contained data archive that included many cities beyond those in the USA. The USA submissions to the CDP, either as originally deposited or as represented in the Nangini et al. archive, were not used in this study. The individual city estimates were not documented sufficiently making alignment of system boundaries difficult to achieve. Indeed, for those overlapping cities between the current study and those archived in Nangini et al., numerical consistency was difficult to achieve. Hence, this study chose to acquire original SRI estimates with available documentation as the SRI dataset upon which to build the comparison reported here.

A limited urban emissions comparison to an older version of the Vulcan data product (version 2.0) was included in Nangini et al. study. However, Vulcan version 2.0 is resolved at a spatial resolution of 100 km^2^ (compared to 1 km^2^ in version 3.0) and represents emissions in the calendar year 2002. The isolation of urban areas in the Nangini et al. study used circular outlines approximating city geographies. Since none of the SRI estimates used in Nangini et al. were aligned with the year 2002, the combination of calendar year mismatch, low-resolution Vulcan results, lack of sectoral detail, differing system boundaries, and the approximate geographical representation make the comparison performed in that paper inadequate as a comparison analysis.

## Data Availability

The complete Vulcan version 3.0 can be downloaded from the Oak Ridge National Laboratory Distributed Active Archive Center (10.3334/ORNLDAAC/1741)^25^. Source data are provided with this paper. All other data and materials are available upon request.

## Source data

Source Data
